# Fusion of green fluorescent protein to the C-terminus of granulysin alters its intracellular localization in comparison to the native molecule

**DOI:** 10.1186/1477-5751-3-2

**Published:** 2004-09-10

**Authors:** Dennis A Hanson, Steven F Ziegler

**Affiliations:** 1 Department of Orthopaedics and Sports Medicine, University of Washington, Seattle, WA 98195 USA; 2 Benaroya Research Institute at Virginia Mason, Seattle, WA 98101 USA

## Abstract

The engineering of green fluorescent protein (GFP) fusion constructs in order to visibly tag a protein of interest has become a commonly used cell biology technique. Although caveats to this approach are obvious, literature reports in which the chimeric molecule behaves differently than the native molecule are scant. This brief report describes one such case. Granulysin, a small lytic and antimicrobial protein produced by cytotoxic lymphocytes, traffics to the regulated secretory system and is subsequently released from cells upon proper stimulus. In an attempt to elucidate mechanisms by which it accumulates in and is released from cytolytic granules, GFP was fused to the C-terminus of granulysin and expressed in an NK cell line. A control construct expressing the native protein was similarly expressed. The data demonstrate that, while the fusion protein is expressed and secreted, its subcellular localization is altered in comparison to native granulysin. Thus, the addition of GFP to the C-terminus of granulysin obscures the signal(s) that cytotoxic lymphocytes use to sort it to the regulated secretory pathway despite its normal biosynthesis and secretion. This example is offered as a cautionary account for other researchers who contemplate using this technology.

## Background

The intrinsically fluorescent protein from the jellyfish *Aequoria victoria*, termed green fluorescent protein (GFP), can be used to visualize dynamic processes in live cells in real time [1]. A fusion between a molecule of interest and GFP is supposed to localize fluorescence to the normal intracellular locale of the target protein. This technology has been used to study the intracellular location and dynamics of many different proteins in many different organisms. Included in this group are proteins that traffic to and ultimately are released from regulated secretory compartments [2]. In this report an attempt was made to use a GFP fusion protein strategy to study the regulated secretory compartment of cytotoxic lymphocytes.

Vertebrate organisms have developed a system of contact-dependent cytotoxicity in order to control tumors and infection. Specialized cytotoxic lymphocytes, T cells and natural killer (NK) cells, function as effector cells in this system [3]. Cell surface receptors on these cells recognize changes to cell surface molecules of transformed or infected cells. Signaling through these receptors initiates target cell destruction. A major method used by these cells to kill targets is the regulated exocytosis of cytolytic granules [4]. The active components of these organelles and mechanisms by which they lead to target cell death have been well studied. However, the underlying molecular mechanisms governing their biogenesis and release remain less well understood. Adaptation of GFP tagging technology to analyze these processes might therefore be of considerable value in elucidating the underlying molecular mechanisms. Thus, GFP was fused to granulysin, a small secreted protein that sorts to and accumulates in cytolytic granules [5,6], and expressed in the functional human NK cell line YT [7]. The YT cell line was utilized in this study because it had previously been stably transfected with native granulysin and shown to properly produce and accumulate the processed product in its regulated secretory compartment [6].

## Findings

**Figure 1:**
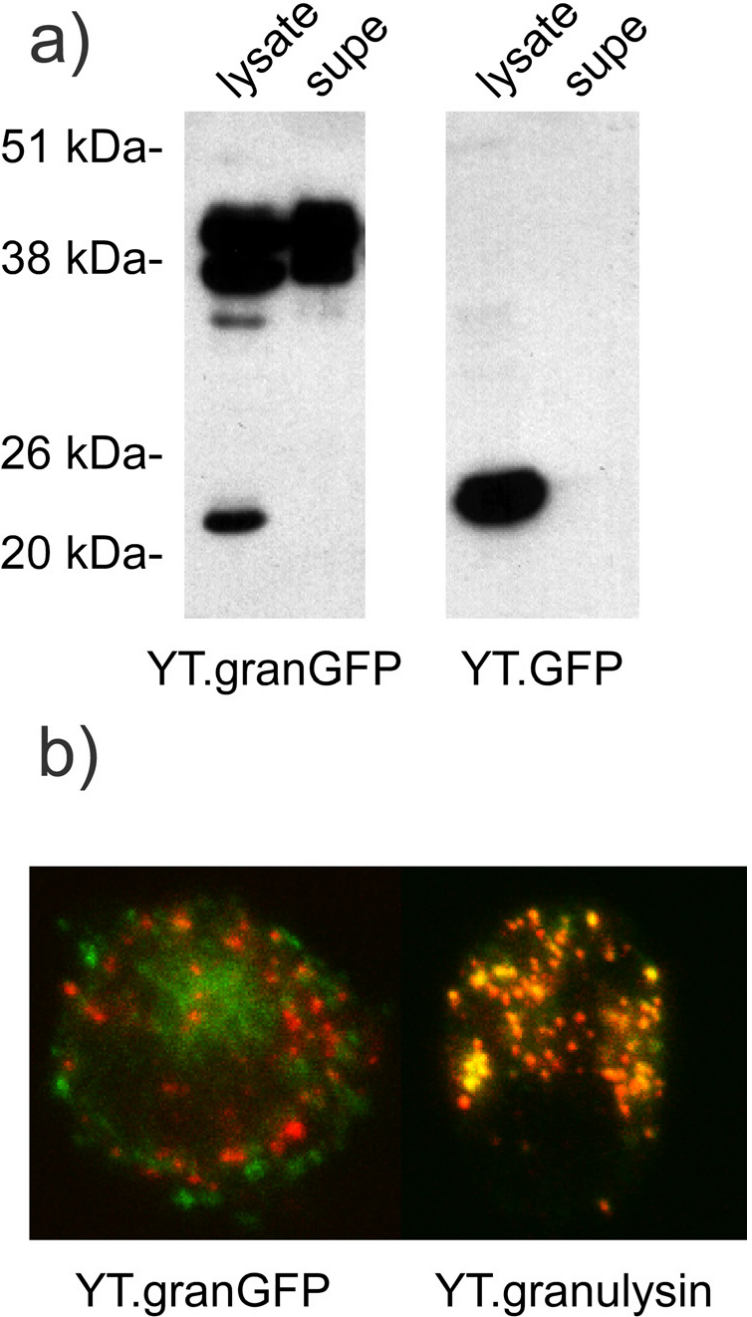
**Granulysin-GFP fusion protein is expressed and secreted but doesn't colocalize with perforin. **a) Immunoblot analysis using anti-GFP sera was performed on cell lysate and cell media supernatant samples of YT cells expressing granulysin-GFP fusion protein (YT.granGFP) and native GFP (YT.GFP). b) Confocal immunofluorescence staining for granulysin (green) and perforin (red) was performed for YT expressing native granulysin (YT.granulysin) and granulysin-GFP fusion protein (YT.granGFP).

Stable transfectant lines for native granulysin, GFP-tagged granulysin, and non-fused GFP were derived using G418 selection. The GFP proteins were first characterized by immunoblot analysis of lysates and cell supernatants probed with antisera to GFP (figure 1a). The cell line transfected with the granulysin-GFP expression construct produces a doublet of proteins in the correct molecular weight range for the fusion protein, with both the cell lysate and supernatant media containing immunoreactivity. No explanation presently exists as to the difference between the two proteins of the doublet. The cell line expressing non-fused GFP, which does not contain a signal sequence, displayed an immunoreactive protein only in the cell lysate and not in the extracellular media. Thus, the granulysin-GFP fusion construct correctly directs the biosynthesis of the chimeric molecule into the secretory pathway. A previous publication demonstrated that a native granulysin transfectant also expresses protein, detectable by anti-granulysin sera, in both lysate and extracellular media fractions [6]. Next, the intracellular localization of native granulysin and granulysin-GFP was analyzed by two color immunofluorescent confocal microscopy using a polyclonal antisera reactive to granulysin and a monoclonal antibody reactive to perforin, a well characterized constituent of cytolytic granules [4] (figure 1b). Significant overlap in the dual staining, as evidenced by the abundant yellow signal, demonstrates that transfected native granulysin co-localizes with perforin in granules. On the contrary, the granulysin-GFP fusion protein displays very little overlap in staining with perforin, indicating that the chimera is altered in its subcellular distribution in comparison to the native molecule. Conclusions to be drawn from these data regarding the mechanism(s) of sorting to cytolytic granules are limited but could suggest that altering the overall biophysical properties of granulysin by the addition of the relatively large GFP moiety negates the information necessary to gain entrance into the correct secretory pathway. However, of perhaps broader scientific significance, the data serve as a striking demonstration of an obvious but seldom published limitation of using GFP fusion proteins as substitutes for the native molecules.

## Materials and Methods

### Cells

The NK cell line YT was transfected via electroporation with the linearized expression construct plasmids of full-length native granulysin-pcDNA3.1, full-length granulysin (res. 1-145)-pGFP and control non-fused pGFP. Stable transfectants were selected and grown in RPMI 1640 + 10% fetal bovine serum containing 1 mg/ml G418.

### Immunoblots

One ml liquid aliquots of log-phase cells were pelleted (supernatant saved), washed in PBS, and lysed in 100 μl of reducing sample buffer. The supernatant was diluted 1:1 (v:v) in 2X sample buffer containing DTT. Ten μl of lysate sample (10% of total) and 10 μl of supernatant sample (0.5% of total) were loaded into wells of a 10% PAGE-SDS gel. After electrophoretic separation, proteins in the gel were transferred to nitrocellulose using a semi-dry blot apparatus. Blots were probed with rabbit antisera specific for GFP followed by peroxidase-conjugated anti-rabbit antibodies. Reactive protein bands were revealed by chemiluminescent detection.

### Confocal microscopy

Log-phase cells were immobilized in wells of a poly-L-lysine coated printed glass slide. After fixation with 4% (w/v) paraformaldehyde in PBS, the cells were permeabilized for 30 minutes in staining buffer [10% (v/v) normal goat serum, 1% (w/v) nonfat dry milk powder, 0.1% (w/v) saponin, in PBS]. Next, samples were incubated for 30 minutes with primary antibodies to granulysin (rabbit antisera) and perforin (mouse mAb) diluted in staining buffer. After washing 3X with staining buffer, samples were incubated 30 minutes with species-specific fluorescent Alexa 488 goat anti-rabbit and Alexa 568 goat anti-mouse secondary antibodies (Molecular Probes). Cells were then washed 3X with staining buffer, then twice with PBS, and glass coverslips mounted using 50% (v/v) glycerol in PBS. Images were collected using a BioRad MRC 1024 confocal imaging system mounted on a Nikon Diaphot inverted microscope.

## Authors' contributions

DAH designed the study, carried out all experimental procedures, and drafted the manuscript. SFZ consulted on the experimental design, provided the resources that allowed the study to be conducted and edited the manuscript. Both authors read and approved the final manuscript.

## References

[B1] Patterson GH, Knobel SM, Sharif WD, Kain SR, Piston DW (1997). Use of the green fluorescent protein and its mutants in quantitative fluorescence microscopy. Biophys J.

[B2] Kaether C, Salm T, Glombik M, Almers W, Gerdes HH (1997). Targeting of green fluorescent protein to neuroendocrine secretory granules: a new tool for real time studies of regulated protein secretion. Eur J Cell Biol.

[B3] Russell JH, Ley TJ (2002). Lymphocyte-mediated Cytotoxicity. Ann Rev Immunol.

[B4] Page LJ, Darmon AJ, Uellner R, Griffiths GM (1998). L is for lytic granules: lysosomes that kill. Biochim Biophys Acta.

[B5] Pena SV, Hanson DA, Carr BA, Goralski TJ, Krensky AM (1997). Processing, subcellular localization, and function of 519 (granulysin), a human late T cell activation molecule with homology to small, lytic, granule proteins. J Immunol.

[B6] Hanson DA, Kaspar AA, Poulain FR, Krensky AM (1999). Biosynthesis of granulysin, a novel cytolytic molecule. Mol Immunol.

[B7] Yodoi J, Teshigawara K, Nikaido T, Fukui K, Noma T, Honjo T, Takigawa M, Sasaki M, Minato N, Tsudo M (1985). TCGF (IL 2)-receptor inducing factor(s). I. Regulation of IL 2 receptor on a natural killer-like cell line (YT cells). J Immunol.

